# Portable Ultraviolet-C Chambers for Inactivation of SARS-CoV-2

**DOI:** 10.6028/jres.126.056

**Published:** 2022-03-18

**Authors:** Shelby Claytor, Roger Campbell, Ashton Hattori, Eric Brown, Christopher Hollis, Max Schureck, Howard Atchley, John Stone, Michael Grady, Benjamin Yang, T. Robert Harris

**Affiliations:** 1 Georgia Tech Research Institute, Atlanta, GA 30318, USA

**Keywords:** disinfection, LEDs, light-emitting diodes, mercury vapor lamps, prototype, PTFE, ultraviolet, ultraviolet-C, UV-C

## Abstract

The goal of this project was to create and optimize the performance of portable chambers for reliable ultraviolet (UV) disinfection of personal protective equipment (PPE) and enable its safe reuse. During unforeseen times of high demand for PPE, such as during the coronavirus disease 2019 (COVID-19) pandemic, caused by the severe acute respiratory syndrome coronavirus 2 (SARS-CoV-2), single-use PPE supply can be quickly depleted. UV radiation has been shown to disinfect materials with high efficacy. This paper reports the design and construction of two 280 nm ultraviolet-C (UV-C) disinfection chambers in the form of portable chambers with 46 cm × 46 cm × 46 cm interior dimensions, one using light-emitting diodes and the other using mercury vapor lamps. This paper summarizes and presents a review of SARS-CoV-2 UV deactivation research during 2020 to 2021. Additionally, this paper discusses efforts to increase the uniformity and overall intensity of the UV-C radiation within the chambers through the installation of a UV-reflective, porous polytetrafluoroethylene (PTFE) material. A calculator prototype was additionally designed to calculate the reduction of SARS-CoV-2 as a result of UV-C disinfection, and the prototype code is presented. The paper describes the selection of UV-C radiation sources for the chambers and the chambers’ mechanical and electrical design, PTFE installation, testing, and safety considerations.

This article was sponsored by Dianne L. Poster, Material Measurement Laboratory, and C. Cameron Miller, Physical Measurement Laboratory, National Institute of Standards and Technology (NIST). It is published in collaboration with the International Ultraviolet Association as a complement to the NIST Workshop on Ultraviolet Disinfection Technologies, 14−15 January 2020, Gaithersburg, MD. The views expressed represent those of the authors and not necessarily those of NIST.

## 1 Introduction

Ultraviolet (UV) radiation disinfection may be used to permit repeated use of personal protective equipment (PPE) designed for single use [1]. This is a critical link in the chain of first-line defense against a contagious outbreak. Single-use PPE items are often disposed of because they are inexpensive and easy to acquire. Under high demand, this may not be an option. In 2019–2020, PPE was under increasingly short supply due to the global coronavirus disease 2019 (COVID-19) pandemic caused by the severe acute respiratory syndrome coronavirus 2 (SARS-CoV-2). Due to scarcity in 2020, face masks, *i.e.*, N95 and FFP2 respirators,^1^1 “N95” is a filter class designation of the U.S. National Institute for Occupational Safety and Health (NIOSH). It is applied to respirators that are at least 95% efficient at filtering NaCl aerosols with particle sizes of mean diameter 75 nm ± 20 nm (NIOSH Procedure No. TEB-APR-STP-0059, 13 December 2019). “FFP2” is a European standard defined by EN149 for protective masks filtering 94% of airborne particles with < 8% inward leakage (European Safety Federation, 26 February 2021). designed for single use were being worn all day and even shared among providers at hospitals and clinics worldwide.

Soft PPE articles, such as N95 respirators, fluid-resistant isolation gowns, and gloves, are issued as single-use articles. Hard plastic face shields see some reuse, with some types intended for long-term use, while other lightweight models are intended to be disposable. COVID-19 hazardous waste daily generation in the United States was estimated to be 8055.03 t [2]. Reports indicate that providers can dispose of more than 50 N95 respirators per day after patient visits [3]. In 2020, as supplies ran low, providers reused, shared, and worked without PPE. While this reuse of PPE is not ideal, it may be the only option that is available in some cases. Given this reality, exposure risk of communicable disease from one user to another can be reduced by disinfecting the reused PPE. PPE can be disinfected a number of ways, including autoclave heat, ozone gas, and chemical disinfectants [4, 5]. Some medical facilities have begun using UV radiation to disinfect equipment contaminated with SARS-CoV-2 [6]. Multiple whole-room devices, including robots, are used to deliver UV sources into unoccupied rooms for safe irradiation [7]. This method has also been suggested as a best practice to disinfect PPE [1]. Chandran *et al.* [8] evaluated two UV-C (200 nm to 280 nm wavelength) disinfection devices for viricidal efficacy on PPE fabric and National Institute for Occupational Safety and Health (NIOSH)–certified N95 respirators through controlled experiments using the H1N1 virus, which is enveloped and is transmitted via the respiratory route similar to SARS-CoV-2. Potential benefits that UV radiation offers over chemical disinfectants is the lack of drying time and absence of potential inhalation of harmful residue, which are factors when fabric N95 respirators absorb chemicals.

The UV-C chambers designed here employed two UV-C sources: mercury (Hg) lamps, a mature technology, and more novel UV-C light-emitting diodes (LEDs). Both technologies were deployed as radiation-source arrays within the chambers, which were designed as portable exposure chambers. UV-A (315 nm to 400 nm) and UV-B (280 nm to 315 nm) radiation wavelengths are not efficacious against SARS-CoV-2, but inferred data and experience of testing similar viruses show that UV-C is efficacious. No peer-reviewed data on the susceptibility of SARS-CoV-2 to UV radiation were available at the start of design; however, there were published data for SARS-CoV (the virus that caused the 2003 SARS coronavirus outbreak), which is closely related (80% genome) to SARS-CoV-2 [9, 10]. The structures of the viruses are the same, and the mechanism of UV-C destruction of the ribonucleic acid (RNA) and protective capsid logically should be highly similar. Blatchley *et al.* [11] presented a recent summary of information to describe inactivation of SARS-CoV-2 by exposure to UV radiation.

## 2 Technical Approach to Develop Portable UV-C Chambers for Inactivation of SARS-CoV-2

This section covers general, optical, thermal and mechanical, and electrical design of the portable UV-C chambers developed in this study. Originally, the desire was to create a UV-C stand that could irradiate medical providers’ PPE while being worn. In this way, upon exiting and entering quarantine zones, the same PPE could be disinfected between patients without removal, thereby reducing the possibility of cross-contamination in an environment where PPE was forced to be reused. It was envisioned healthcare providers would exit a hospital room, stand in front of the device for a period of time, and then move on to the next patient. This approach was abandoned due to concerns about skin and eye exposure to UV-C and potential health risks. The UV-C stand concept would not allow for coverage of the skin, for example, exposed skin above a face shield, nor eyes without protective coverings. It also was challenging to ensure complete surface disinfection of three-dimensional structures within a chamber, due to shadowing or indirect illumination.

The selected design alternative was a portable exposure chamber with a mass under 14 kg and extendable trolley handle. In this design, PPE could be exchanged in and out of the chamber with treatment inside a closed chamber. The portable nature of the chamber would be suitable for use in ambulances, in pharmacies, or in urgent care centers. Customizable aluminum 80/20 T-slot frames were considered for the prototypes, but ultimately ruggedized ridged plastic boxes were selected as the chassis. The size of the chamber was dictated by the desire to fit at least one hard plastic face shield inside. The finished exposure chambers could fit one to two face shields, depending on their size. These items rest on a wire screen. Internal dividing walls were used to mount UV-C radiation sources and to retain electrical components. The suggested use of this chamber is as a supplement, rather than a replacement, to all recommended procedures, for example, if there is currently preexisting policy in place using chemicals or heat.

A pair of Aquisense PealLab Micro UV-C LEDs and OSRAM Germicidal Puritec HNS 4P 16 W Hg lamps were purchased.^2^2 Certain commercial equipment, instruments, or materials are identified in this paper to foster understanding. Such identification does not imply recommendation or endorsement by the National Institute of Standards and Technology, nor does it imply that the materials or equipment identified are necessarily the best available for the purpose. The prototype chambers for each type of UV radiation source were developed at the Georgia Tech Research Institute (GTRI). All testing and evaluation of telemetry, timers, proximity sensors, and UV dosimeters were conducted at the GTRI. Solid-state LEDs should be more rugged than the glass-tube (Hg) sources for environments with shock and vibration. Standard inverters providing at least 300 W can be used for both chambers for use with automobile power.

### 2.1 Optical Design

A timer was incorporated to ensure sufficient UV dosing of items for disinfection. Previous UV-C disinfection chambers have used UV radiation detector photodiodes as feedback to control the intensity of the UV radiation sources. For example, intensity meters have been used for water-disinfection applications because, depending on turbidity, dissolved Ca and Fe highly attenuate UV-C. The intensity meters can dim UV output by adjusting voltage or duty cycle to optimize lamp lifetime, power consumption, and disinfecting power [12]. UV intensity or power sensors can also be used to determine LED or Hg lamp health along with age [13]. The same UV sensors can be used for air or water applications but will be normalized for the medium. Solids dissolved in water are UV absorbent. The prototypes presented here did not include UV power meters, but these could be incorporated in order to dynamically adjust exposure time or warn of incomplete sanitization. Important considerations affecting UV intensity on an irradiated object are distance from the source, angle of incidence, intensity, and usage age of the radiation source. Exposure time has a safety factor built in so that the target dose is exceeded.

Common recommended dosages for treating SARS-CoV in a nonaqueous environment range from 1 J/cm^2^ to 2 J/cm^2^ [14–19]. Erring on the side of caution, a 2 J/cm^2^ dosage was used here. It is important to note that some studies apply UV-C to aqueous solutions, which require much higher dosages for disinfection. In the case of disinfecting PPE, the recommended dosages from aqueous studies do not apply. The most common metric used when characterizing UV disinfection is dosage, commonly in joules per square centimeter (J/cm^2^). In order to convert from UV intensity to dosage, the time of radiation exposure is required. From there, a calculation is used, as seen in the unit analysis below:

$Dose\;\left[\frac{J}{cm^{2}}\right]=Intensity\;\left[\frac{W}{cm^{2}}=\frac{J}{s∙cm^{2}}\right]\times Time[s]$ (1)

If the required dosage for disinfection and the UV-C source intensity are known, then the time of disinfection can be calculated, according to the desired level of disinfection, *i.e.*, 2 log_10_, 3 log_10_ reduction, *etc*.^3^3 “log_10_” reductions are calculated as log_10_ (*N*_0_/*N*), where *N*_0_ is the initial value, and *N* is the final value; 2 log_10_ units refer to a 99% reduction. Disinfection times shown in Table 1 were based on measurements of the sources. Table 1 provides a listing of UV-C LED intensity measurements from this study. The power measured by the UV power meter is listed along with the intensity using the sensor area.

**Table 1:** UV-C LED intensity measurements.

**Configuration**	**Power at 7 cm Height (mW)**	Corresponding Intensity ($\frac{mW}{cm^{2}}$ for** 1 cm Diameter)**
**Lid open**	1.502	1.9
**Lid closed**	1.200	1.5

Assuming two identical UV-C sources, no cover, and similar conditions, a $2\frac{J}{cm^{2}}$ dose would require a disinfection time of

$Time=\frac{2\;J/cm^{2}}{2\times 1.912\;mW/cm^{2}}=523\;s=8.7\;min$ (2)

**Fig. 1:**
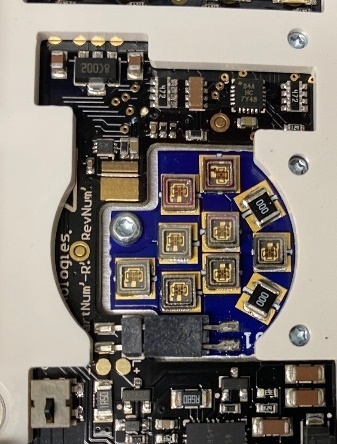
UV-C LED sources visible on printed circuit board (PCB) with cover removed.

Two separate chambers were designed in order to compare and contrast the performance and chamber design considerations arising from source differences; one used UV-C LEDs, and the other used Hg lamps. UV-C LED sources were installed in the top and bottom of the chamber. Figure 1 shows the UV-C LED components of the source. The Hg lamps were installed in the same configuration as the UV-C LED sources, so that the top and the bottom of items could be illuminated.

Internally, for both UV-C LED and Hg lamp chambers, the materials used were brushed aluminum, as internal bent sheet metal structure, and a UV-reflective, porous polytetrafluoroethylene (PTFE) material (as PTFE walls). A polished aluminum surface may allow for higher internal reflection within the chamber. Later in development, all internal brushed aluminum was covered by PTFE sheets. High internal reflectivity will increase full coverage exposure of the contaminated PPE to optimize the breadth of exposure and decrease undesirable masking and shadow effects. Masking is more difficult to overcome when an object opaque to UV wavelength is directly touching and obscuring the surface of the PPE, such as a head strap or another respirator. To achieve bulk reflectivity of UV-C, PTFE thickness is recommended to be of at least 6.35 mm, with further improvement up to 12 mm. Thinner PTFE sheets with adhesive backing acquired from a hardware supplier were used in these prototypes to reduce cost while retaining some beneficial reflective effects. The reflective properties were compared to a porous PTFE material, which has a structure allowing it to reflect UV radiation as well as thicker solid PTFE. PTFE, aluminum, and transmissive glass source tubes and windows must all be cleaned with isopropyl alcohol (IPA) for highest performance. Oils imparted due to handling degrade UV-C reflection and transmission through absorption.

### 2.2 Mechanical and Thermal Design

**Fig. 2:**
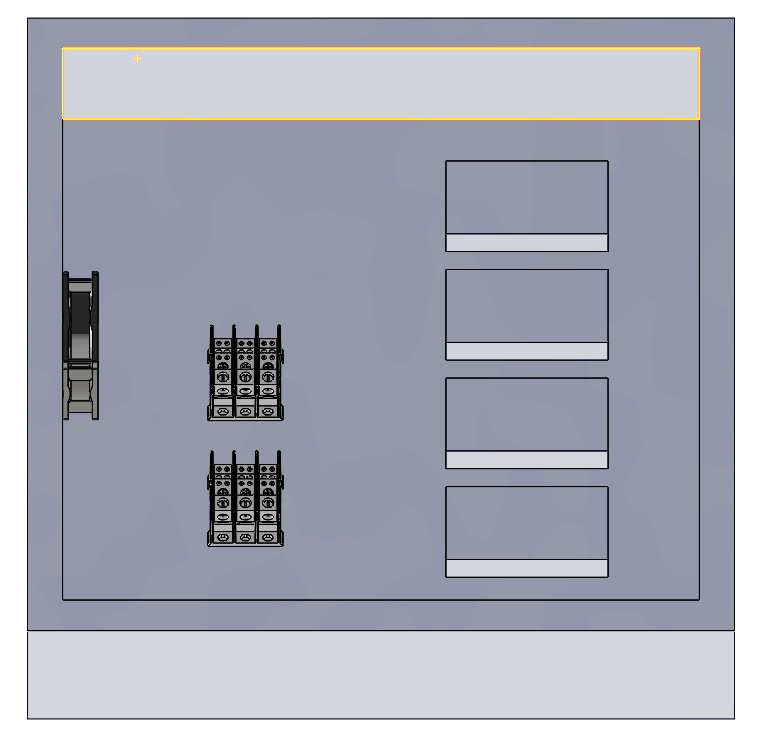
The spacing and layout of the 45 cm × 45 cm × 8 cm fabricated sheet metal, which was used to fit all of the electrical components under the divider space.

**Fig. 3:**
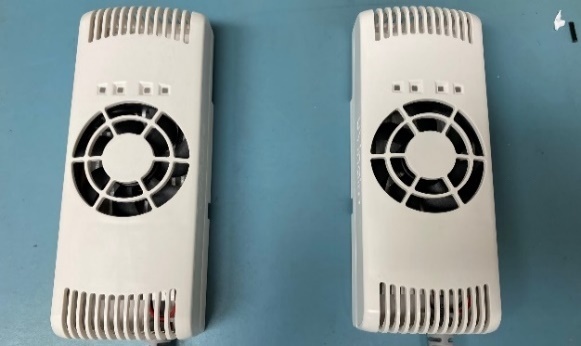
UV-C LED housings with integrated heat sinks and fans irradiate targets from the top and bottom. Hot air is exchanged through vents in the chassis.

Electrical component spacing and attachments were laid out for space planning as shown in Fig. 2. Safety interlocks were fabricated so that the system could not be turned on without the lid being completely shut, thereby avoiding unintentional exposure to skin and eyes. The UV-C LED housings with integrated heat sinks and fans are shown in Fig. 3.

**Fig. 4:**
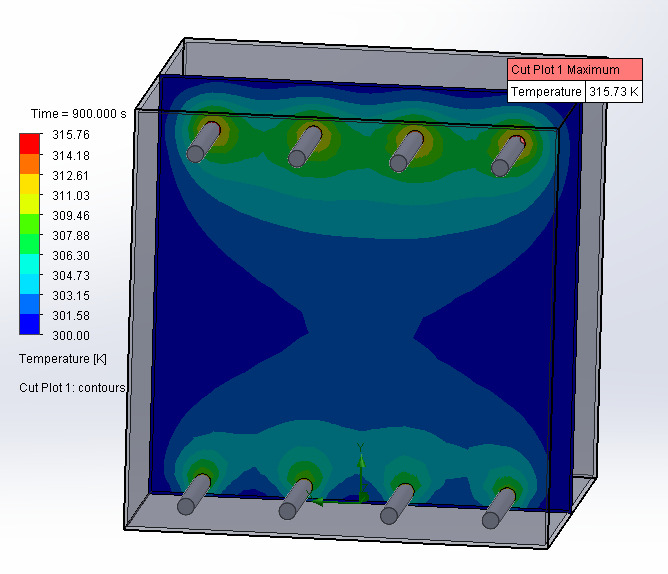

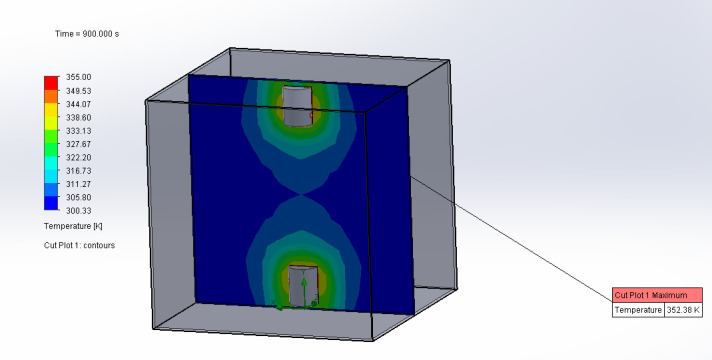

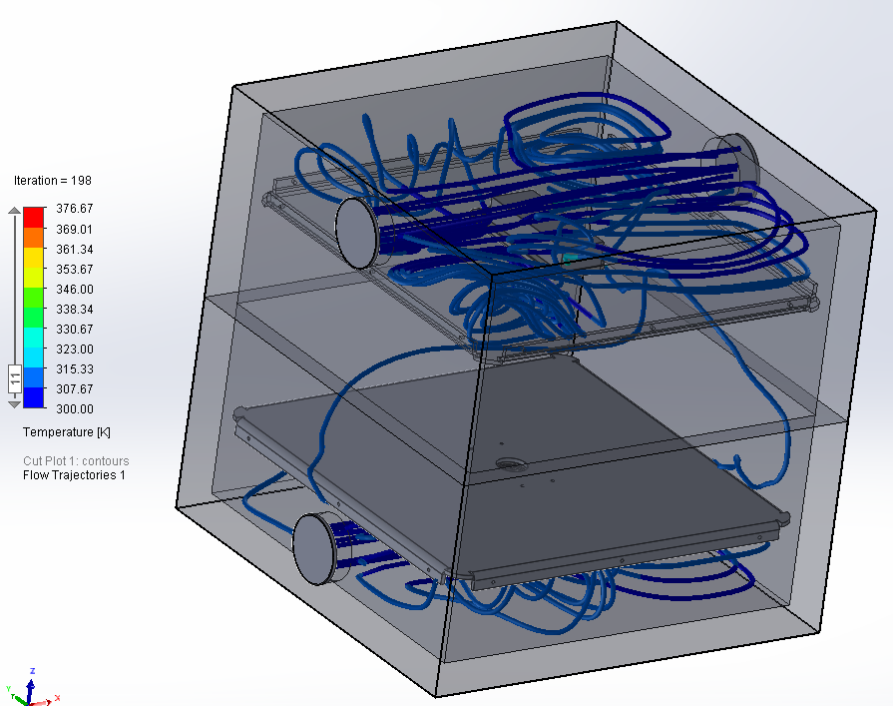
Thermal simulation results using Solidworks Flow Sim Thermal compared for the Hg lamp (top) and LED (middle) UV sources to determine fan and air venting requirements. Airflow simulation resultant from fans to determine the need for exhaust ports is shown (bottom). The LEDs heat sources were simulated using Solidworks with cooling fans at steady state.

Thermal simulation using Solidworks Flow Sim Thermal was used to determine the fan requirements for both the Hg fluorescent lamps and the LED chambers. Results of the simulations are displayed in Fig. 4. Fluorescent lamps and ballasts do not include integrated cooling and normally rely on passive convection cooling in a nonenclosed environment. For the UV appliance models, which are enclosed chambers, a 27 °C ambient temperature was used based on an ambulance with air conditioning in the summer. Adiabatic walls were used as boundary conditions for conservative measure, so it was assumed there was no natural heat loss in the chamber. Input heat used for the ballasts was 1 W each based on 45 W maximum input with a >0.98 power factor. Input heat for the fluorescent bulbs was 16 W assuming 0% efficiency at 16 W heat each. For the LED alternative chamber, the input power for the LEDs was 40 W with 5% efficiency thus 38 W of heat was used for each source in the simulation.

The results of the simulations demonstrated that no fans were needed to cool the Hg lamps and ballasts when vents were cut in the appliance chassis. Similarly, no fans were required to cool the LEDs if a passive vent was present. However, cooling fans for UV-C LEDs helped to ensure consistent operating temperatures while mitigating the risk of overheating. The thermal design recommendation is for passive cooling above the top divider, with fans installed below the bottom divider for prevention of overheating. Hg lamps have a minimum temperature requirement for effective output intensity and are not easily damaged by high temperatures. LEDs are smaller, have a higher current density, and have a higher heat flux than tube lamps. LEDs age over time at high temperature, and their output intensity is reduced by high temperature [12].

In the case of the Hg lamp assembly, the lamps, ballasts, and distribution blocks were placed below the target surface. Similarly, for the UV-LED assembly, the DC power adapters were installed below the lower divider, so the fan installation in the lower section was beneficial for temperature control. Some air flow between the lower and upper divider sections was available for additional cooling.

**Fig. 5:**
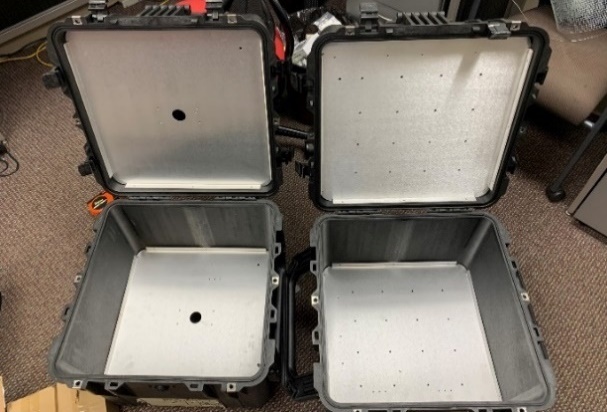
The rugged box chassis with 46 × 46 × 46 cm interior dimensions and sheet metal dividers installed was designed for both LED and Hg lamp germicidal UV sources.

Figure 5 shows the two chassis boxes used to created UV disinfection chambers. Bent sheet metal dividers were fabricated to provide the internal structure used to mount and store electrical components. The metal dividers were made by measuring the interior of the chambers, using computer-aided design drafting, and adding mounting holes. The components mounted on the dividers were the electrical distribution blocks, ballasts, and lamp holders. A corner pass-through allowed for top to bottom power supply connection. Sheet metal was cut and bent at the GTRI machine shop.

**Fig. 6:**
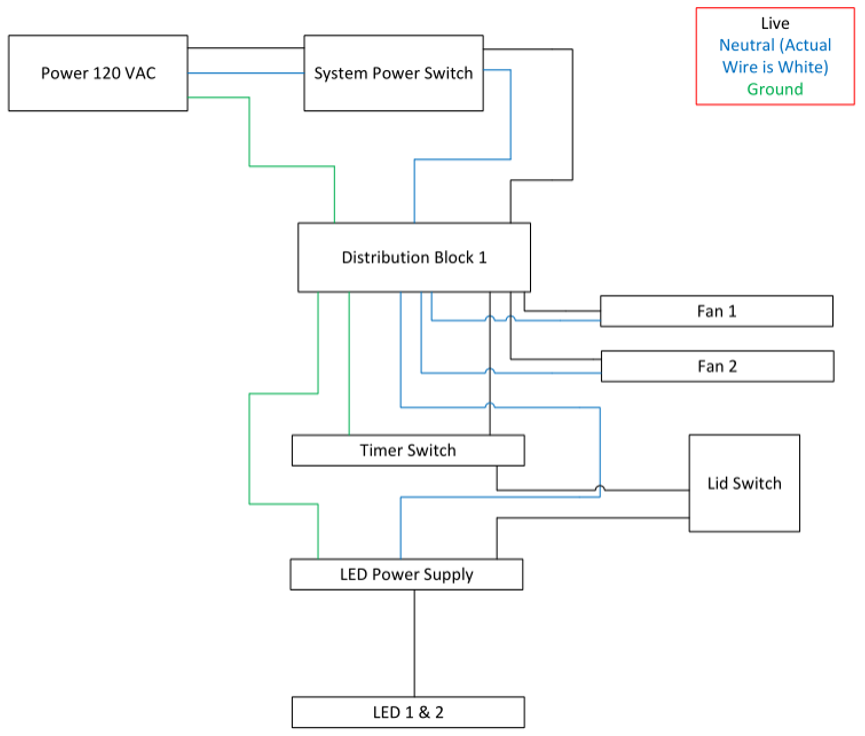

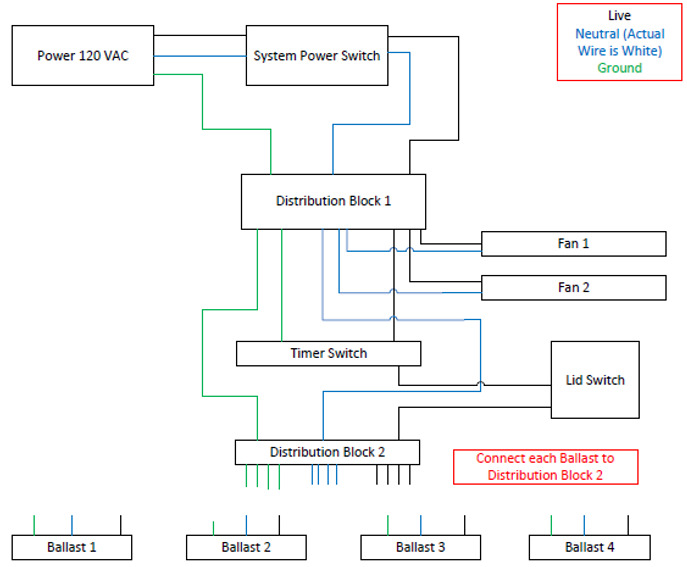
The UV-C LED source (top) and Hg lamp source (bottom) electrical system diagram with safety interlocks to prevent unintentional exposure without the lid in place. A timer ensured correct dosage times based on measurements for a 6 log_10_ reduction of virus.

### 2.3 Electrical Design

Wiring was completed for each disinfection chamber according to the wiring schematics as shown in Fig. 6. The bottom divider space contained the power cord with a waterproof gasket, two ballasts in the case of the Hg lamps system, power supply in the case of the LED system, and one or two distribution blocks depending on the system. Power supplies were fused at the input for safety. The top divider section contained the chamber power switch, UV enable switch, associated indicator lights, and timer switch. Future iterations will compute the proper exposure time based on the intensity and required dosage. The electrical timer would be replaced by electronic timers and controllers.

### 2.4 PTFE Installation

**Fig. 7:**
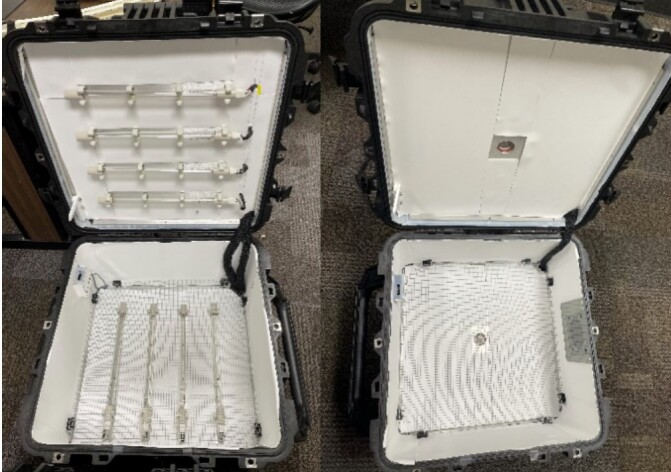
The Hg lamp (left) and UV-C LED (right) chambers with reflective PTFE installed on the top, bottom, and side walls of each chamber.

After initial intensity measurements were taken, POREX Virtek® PMR10 porous PTFE was installed on the side walls of each box, avoiding any wiring and the tinted window on the side of the UV-C LED system. This PTFE material is optimized specifically to reflect UV radiation. After intensity measurements were taken in each box with this configuration of PTFE, additional PTFE was installed on the top and bottom of each box. Panels of PTFE were placed across the top and bottom, avoiding direct contact with each radiation source while attempting to eliminate gaps between each panel that would expose the brushed aluminum beneath. Intensity maps were created by taking measurements over a 5 min period with a Newport Power Meter 2936-R at 12 locations on the metal mesh within each chamber. The measurement points were located at 0, 15 cm, 30 cm, and 45 cm across the box, and at 0, 23 cm, and 45 cm along the length of the box. Figure 7 displays each chamber with PTFE on the sides, top, and bottom walls. It should be noted that the PTFE used in this case was porous and had a thickness of 0.75 mm. Pathogens could potentially enter and remain in the porous material.^4^4In a 2013 investigation by Lopez et al. [20] on the effect of low and high relative humidity on fomite-to-finger transfer efficiency of Escherichia coli, Staphylococcus aureus, Bacillus thuringiensis, MS2 coliphage, and poliovirus 1 from several common inanimate surfaces (fomites), “nonporous surfaces had a greater transfer efficiency (up to 57 %) than porous surfaces (˂6.8 %) under low relative humidity, as well as under high relative humidity (nonporous, up to 79.5 %; porous, ˂13.4 %).”  Due to the PTFE pore size, bacteria and viruses cannot enter the material, with the vendor reporting >99.99 % viral filtration efficiency ^5^5Personal communication, Porex Corporation, March 2022.. If contaminants remain on the surface of superficial pores, it is expected these would be irradiated. 

## 3 Technical Results

At the time of sourcing components, germicidal Hg lamps were already in short supply and selling out from many vendors. With choices of type limited and increasing prices, it was decided to also develop an LED alternative. UV-C LED packages integrated with a thermal management heat sink, fan, and power supply were purchased from Aquisense. Rather than requiring a ballast, the LED housings use small DC power adapters, which were easily installed into the chassis. The units feature temperature monitoring, will automatically shut off if overheated, and emit radiation at 280 nm.

The Hg lamps acquired consisted of a soda lime glass sleeve, which does not filter some of the broader spectra of UV emitted by the Hg lamp [21]. UV radiation from the 185 nm Hg line can generate ozone (O_3_), which is filtered by the glass used by this tube model. A 254 nm germicidal line is also produced. Overall, the lamps create more total heat than the LEDs, which is added to that created by the chassis fan. The associated ballasts also create heat.

Some detractors for the use of the Hg sources include toxicity and environmental contamination. The lamps on the bottom of the appliance were guarded by the wire target shelf that holds items to be disinfected, but the lamps in the top lid were left exposed in the prototype. They could accidentally be touched and broken, contaminating the space with breathable Hg vapor. A pellet of Hg amalgam slides freely in the sleeve, which equalizes the pressure of Hg vapor within the lamp. This provides some limit of exposure, yet it still contaminates the environment and requires special disposal and handling. Furthermore, the Hg lamps take time to warm up and reach full intensity by 45 s, which was measured in our laboratory. This is a feature of all Hg lamps, especially spot and pellet lamps like those used here. As such, due to the warm-up time, it is possible that longer exposure times may be required with the Hg lamp system to achieve the appropriate safe dosage relative to the UV-C LED system, which does not require a warm-up time, despite the greater intensities achieved with the large number of Hg lamps.

Intensity maps were created for each chamber in three states: initially (with no reflective PTFE present), with PTFE on the side walls, and with PTFE on the sides, top, and bottom. The percent increase in the average intensity within each chamber resulting from the addition of the porous PTFE was determined from measurement data. Additionally, the percent difference of intensity from the highest intensity point to the lowest intensity point inside each chamber was also determined in order to measure the uniformity of the UV-C radiation.

The average intensity in the Hg lamp chamber exhibited a 42% increase when PTFE was present on the sides of the box compared to the initial intensity measurements. When PTFE was present on the top, bottom, and sides of the box, the average intensity increased 115% from the initial intensity measurements. Initial measurements showed a 69% intensity difference from the highest to the lowest intensity points in this chamber. Measurements taken with PTFE on the sides of the box showed a 44% internal decrease in intensity difference, and that stabilized around a 47% difference with PTFE present on the sides, top, and bottom of the box. The Hg lamp chamber improved significantly both in the average intensity of the UV radiation and the uniformity of the radiation intensity when the porous PTFE was installed on its sides and subsequently on the top and bottom.

**Fig. 8:**
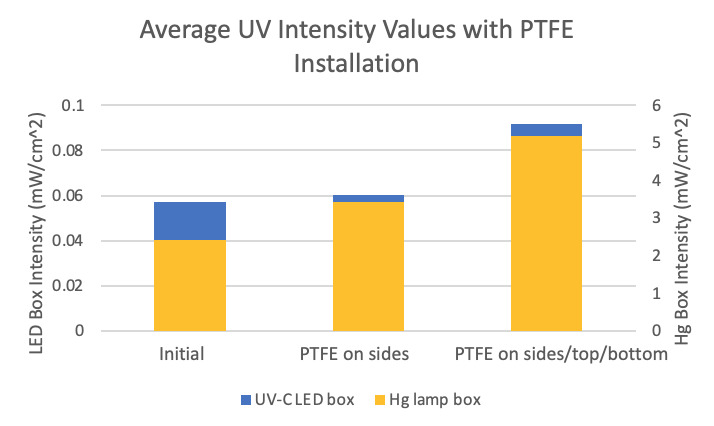
Gain in intensity with the installation of porous 0.75 mm PTFE relative to nonporous PTFE.

**Fig. 9:**
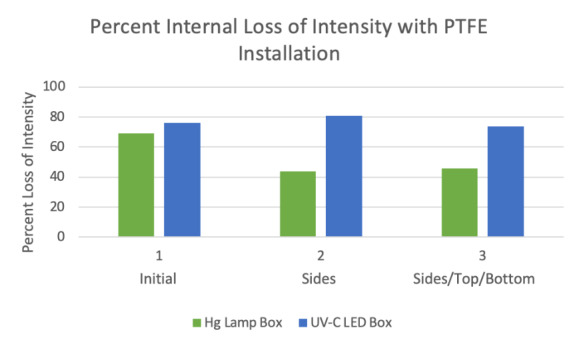
Variation in intensity across the plane of the chamber with installation of porous 0.75 mm PTFE.

The average intensity in the LED bulb chamber increased by 6% from initial measurements when reflective PTFE was installed on the side walls of the box. It increased by 61% from initial measurements with PTFE present on its sides, top, and bottom. Initial measurements showed a 76% intensity difference from highest to lowest intensity locations within the box. The percent difference was 80% and 74% when PTFE was installed on the sides and on the sides, top, and bottom of the chamber, respectively. This chamber only improved in the average intensity of UV radiation when PTFE was added. The intensity uniformity gradient was not significantly different. This is likely due to the design. The radiation sources in this chamber were two small circular LED UV windows, placed on the center top and bottom of the box. In the case of the Hg lamp system, the Hg lamps were oblong, and there were four lamps placed along the length of the top and bottom of the box. There was also a tinted window on a side wall of the LED UV chamber, through which radiation intensity could have been lost. This likely caused significantly greater uniformity of radiation as well as intensity of radiation overall, which was evident in the measurement data. Figures 8 and 9 display the percent change in average intensity with PTFE installation as well as the percent internal difference. There was little long-term drift, and intensity was measured to be stable over time.

**Fig. 10:**
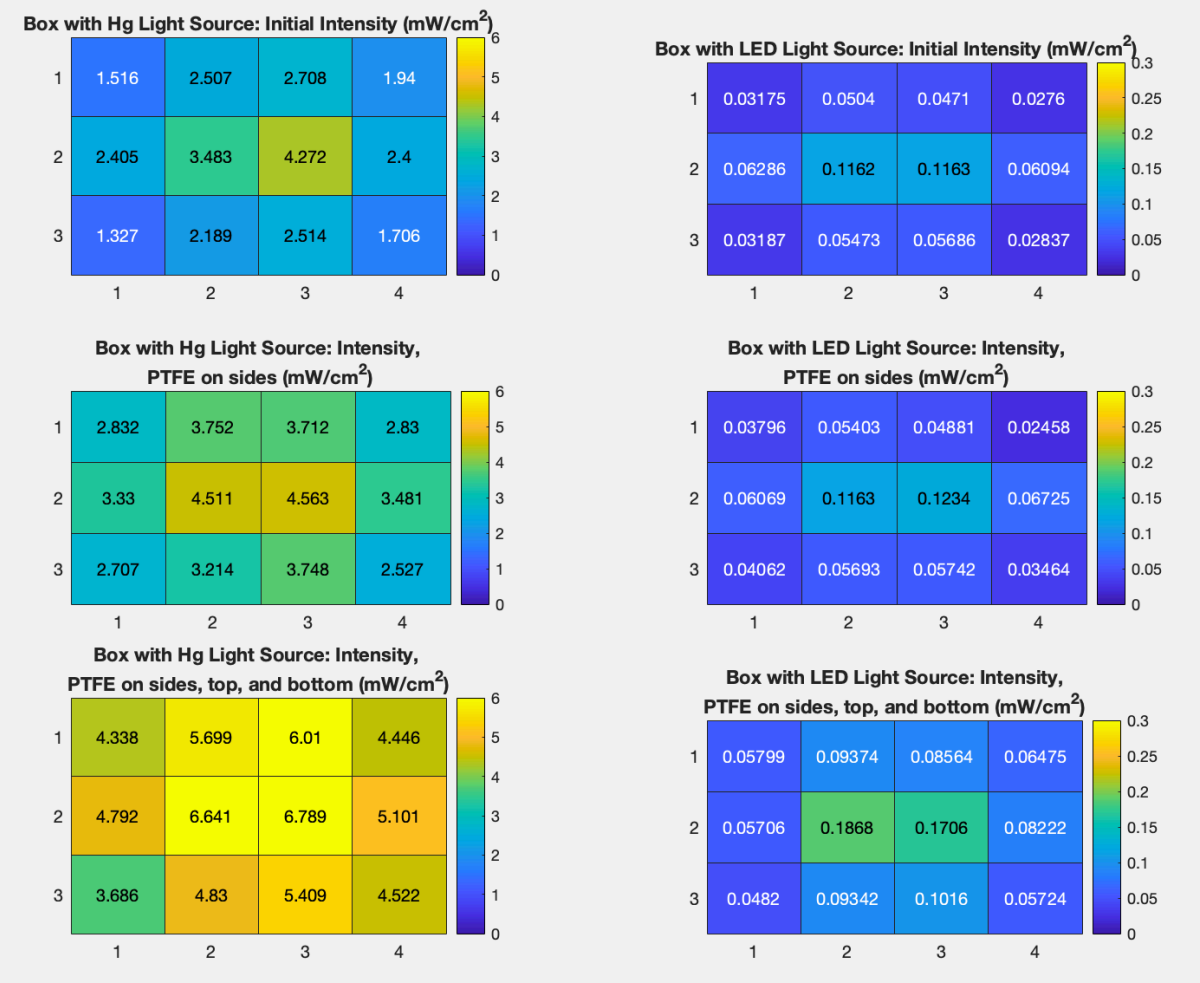
Maps displaying the intensity of radiation at 12 locations in each UV chamber initially and with the addition of the reflective, porous PTFE.

Intensity maps were created to illustrate these measurements within each chamber and are displayed in Fig. 10. These maps provide data to determine the effect that an applicable object, such as a piece of PPE equipment, would have on the radiation intensity inside a UV chamber. The boxes had reflective PTFE previously installed on the top, bottom, and on the side walls. In the Hg lamp chamber, the average intensity measurements taken with the face shield present showed a 34% decrease from measurements taken without the PPE present. The difference experienced internally from points of high to low intensity in the box also increased by 16%. A similar effect was observed in the UV-C LED box. In this case, the average intensity decreased by 78%, and the internal difference increased by 9%.

**Fig. 11:**
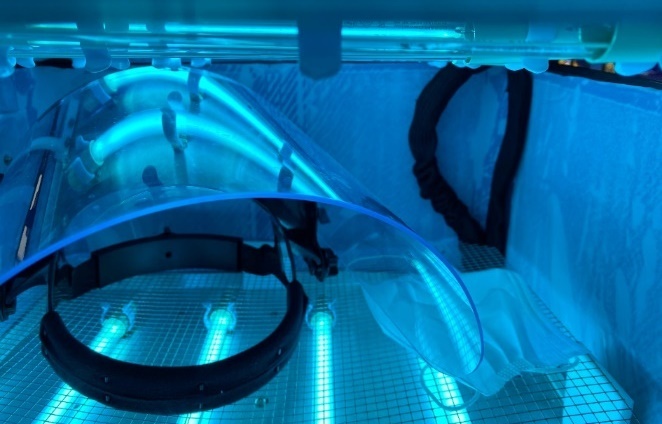
The finished appliance irradiating a face shield and mask.

**Fig. 12:**
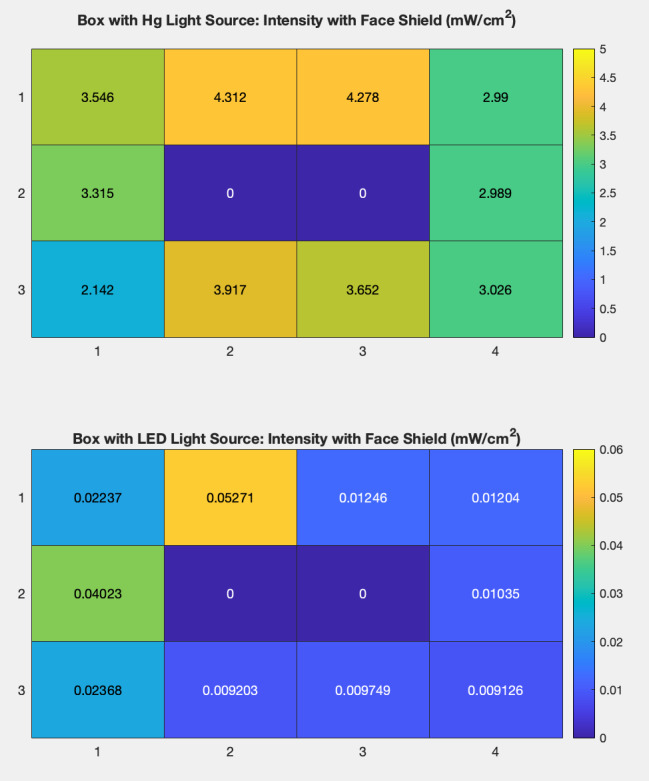
Maps displaying the UV radiation intensity surrounding a face shield (indicated by 0) within each UV chamber.

**Fig. 13:**
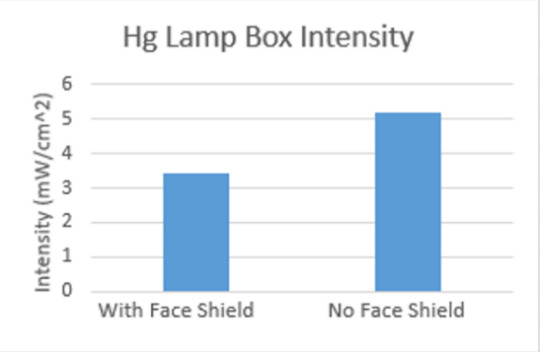

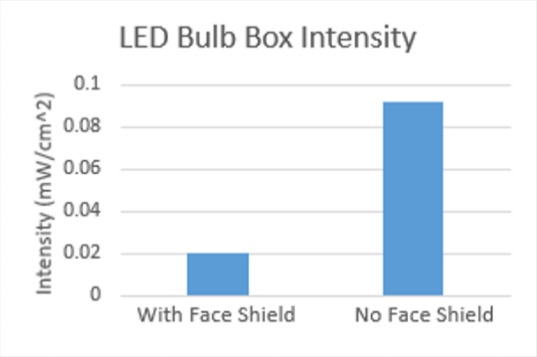
Graphs showing the average intensity in each UV chamber when a face shield was present inside the chamber compared to the intensity measured in an empty chamber.

A face mask being irradiated by UV is shown in Fig. 11. Intensity measurements were also taken in each box when a face shield was placed inside, as represented by Fig. 12. Figure 13 shows these data as a function of the average intensity in each chamber that occurred from the presence of the face shield. Overall, the intensity values measured with the face shield present show a significant lack of uniformity. This may be due to differences in reflectivity and radiation absorption on different regions of the face shield. Alternatively, the face shield could have physically blocked UV radiation from reaching the power meter during measurement. The presence of objects within the UV chamber can significantly alter the intensity of radiation in locations surrounding the object. Tangential irradiation incidence will decrease radiation intensity on objects. Color change–indicating UV dosage test strips adhered to the test mask showed levels beyond *E. coli* disinfection levels on all sides.

### 3.1 UV Chamber Technical Calculator

**Fig. 14:**
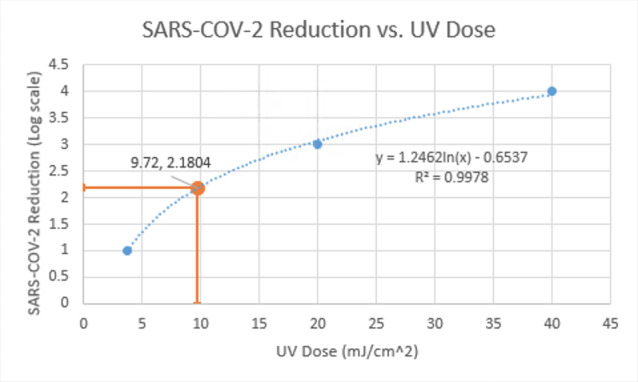
A demonstration of example inputs into the calculator script and the subsequent output. The given inputs indicate that an Hg lamp system is in use with reflective PTFE on the side walls of the chamber. The UV intensity is listed at 0.5 mW/cm^2^, for an exposure time of 15 s. This would result in a 2.1804 log_10_ reduction of SARS-CoV-2, which corresponds to approximately 99% inactivation. A plot of the logarithmic regression relating UV dose to SARS-CoV-2 reduction is also shown, with the example script inputs indicated.

A notional MATLAB calculator script was designed to determine the reduction of SARS-CoV-2 that would occur based on several user inputs characterizing the UV disinfection chamber and making use of a literature-based regression function for SARS-CoV-2 (see Fig. 14). This calculator allows the user to evaluate and make improvements to their UV disinfection chamber to achieve the appropriate level of inactivation of the SARS-CoV-2 virus. All calculations implemented by the script run under the assumption that the UV chamber in use was designed in a similar manner to the chambers discussed in the previous sections of this paper. When the script is run, the user will input several pieces of information relating to their UV chamber. This includes the type of UV radiation source in use (Hg or LED radiation system), the presence and location of UV-reflective PTFE within the chamber, the exposure time in seconds, and UV radiation intensity in milliwatts per square centimeter. This calculator considers only the middle of the chamber; however, future work can add uniformity functionality to the entire volume.

The intensity of the UV radiation is first multiplied by a factor that reflects the radiation system in use as well as the presence and location of UV-reflective PTFE within the chamber. These factors were determined from the percent increase in average intensity resulting from each addition of PTFE within the UV chambers in this study. If no reflective PTFE is present, the intensity value remains the same as the user’s initial input. Then, the adjusted intensity value is multiplied by the exposure time in order to determine the total dose of UV in millijoules per square centimeter.

The UV dose is then input into a logarithmic regression that quantifies the relationship between UV dose and reduction of SARS-CoV-2. A literature review [22—26] was conducted to determine the range of UV doses that lead to increasing levels of SARS-CoV-2 inactivation (based on a log_10_ scale of reduction). This range was used to create the logarithmic regression that appears in the calculator script. This regression has a high potential for inaccuracy due to the approximate nature of the literature review, but improvements could be made with more thorough range-finding techniques. The reduction level of SARS-CoV-2 determined from the UV dose is output to the user as a percentage for ease of understanding. Any reduction greater than 4 log_10_ is displayed as a virtually complete reduction of SARS-CoV-2. The code listing below shows an excerpt from the script, in which the UV dose and SARS-CoV-2 reduction are calculated from the adjusted intensity value. Additionally, Fig. 14 shows an example input to the code, the resulting SARS-CoV-2 reduction output, and a graph of the logarithmic regression used for calculating the reduction.

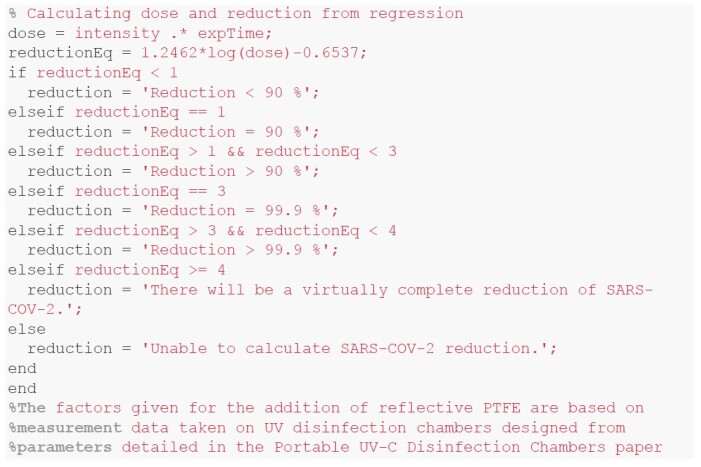



The next block of code from the MATLAB calculator script determines the UV dose and expected SARS-CoV-2 reduction based on an intensity value adjusted for the reflective PTFE present within the UV chamber.

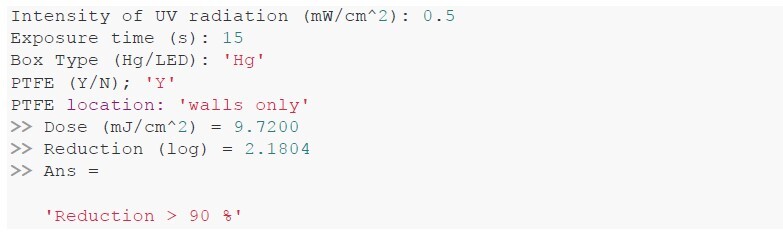



## 4 Conclusion

Two UV disinfection chambers were designed, developed, and fabricated for the inactivation of SARs-CoV-2. The design process is described in this paper. Proper wavelengths, exposure intensities, and times for viral inactivation were calculated and utilized based on existing literature, understanding that confirmatory pathogen reductions would be required prior to clinical deployments of these chambers. Mechanical considerations such as a safety interlock and vertical suspension of contaminated targets on a thin wire screen were described. The procedure of installing UV-reflective PTFE inside the chambers, and its positive effect on the intensity and uniformity of UV radiation, was discussed. The portable UV chambers are currently available for use and testing within the community for feedback and design iteration.

This paper also describes the creation of a MATLAB calculator script that determines an approximate percent reduction of SARS-CoV-2 based on the input UV chamber information. This script is intended to assist users of UV disinfection chambers in proper operation of the chambers to achieve appropriate levels of inactivation of the SARS-CoV-2 virus. While this initial script has a high potential for inaccuracy due to the approximate nature of the inactivation ranges input from literature, improvements could be made with more thorough range information.

Lessons learned from the development of the portable UV chambers include safety factors to avoid any possible skin exposure to UV during assembly and testing. Improvements needed for the chambers include decreasing their size and weight. The addition of UV-reflective materials along the walls of each chamber and the elimination low-UV-intensity zones will also improve the performance of the chambers for viral inactivation. While there is difficulty in fitting electrical components within the limited space for an effective dose, a more compact and lighter chamber can be useful in more applications. It is desirable to provide a cost effective solution while considering exposure time. For example, if longer exposure times can be afforded for an application, then radiation sources with lower intensity can be used, while decreasing chamber cost. Certain applications will benefit from shorter exposure times. Improving the performance of the chambers could decrease necessary exposure times.

More advanced electronics and components with recipes on a programmable controller are being developed along with a UV sensor that can monitor UV source health and intensity and adjust exposure time accordingly.

## References

[1] Geldert A, Balch HB, Gopal A, Su A, Grist SM, Herr AE (2021) Best practices for germicidal ultraviolet-C dose measurement for N95 respirator decontamination. Journal of Research of the National Institute of Standards and Technology 126:126020. 38469452 10.6028/jres.126.020 PMC10046750

[2] Haque MS, Uddin S, Sayem SM, Mohib KM (2021) Coronavirus disease 2019 (COVID-19) induced waste scenario: A short overview. Journal of Environmental Chemical Engineering 9(1):104660. 33194544 10.1016/j.jece.2020.104660 PMC7648514

[3] Personal conversation, multiple nurse practitioners at Emory Healthcare, March 2020.

[4] Ebeling D, Werner J, Erbe J, Ebeling S, Miao Q, Ebeling A, Sanford L, Boinski C, Salous B, Scheunermann A, Peaslee D, Ploense L, Ploense R, Setter E, Findlay M, Meulendyk, B, Lee M, Patel, V, Stetter JR (2021) Validation of a low-cost on-demand compact ozone Covid-19 sterilization chamber. *ECS Meeting Abstracts* MA2021-01:2024.

[5] Saini V, Sikri K, Batra SD, Kalra P, Gautam K (2020) Development of a highly effective low-cost vaporized hydrogen peroxide–based method for disinfection of personal protective equipment for their selective reuse during pandemics. Gut Pathogens 12(29):1–11. 32572338 10.1186/s13099-020-00367-4 PMC7303439

[6] Julig C (2020) Thompson Valley EMS uses ultraviolet light to disinfect ambulances from coronavirus. *Loveland Reporter-Herald.* Available at https://www.reporterherald.com/2020/03/31/coronavirus-thompson-valley-ems-uses-ultraviolet-light-to-disinfect-ambulances-due-to-covid-19/

[7] McGinn C, Scott R, Donnelly N, Roberts KL, Bogue M, Kiernan C, Beckett M (2021) Exploring the applicability of robot-assisted UV disinfection in radiology. Frontiers in Robotics and AI 7:590306. 33501347 10.3389/frobt.2020.590306 PMC7815819

[8] Chandran KM, Ramamurthy PC, Kanjo K, Narayan R, Menon SR (2021) Efficacy of ultraviolet-C devices for the disinfection of personal protective equipment fabrics and N95 respirators. Journal of Research of the National Institute of Standards and Technology 126:126023. 36475082 10.6028/jres.126.023 PMC9681524

[9] Darnell MER, Subbarao K, Feinstone SM, Taylor DR (2004) Inactivation of the coronavirus that induces severe acute respiratory syndrome, SARS-CoV. Journal of Virological Methods 121(1):85–91. 15350737 10.1016/j.jviromet.2004.06.006 PMC7112912

[10] Kariwa H, Fujii N, Takashima I (2006) Inactivation of SARS coronavirus by means of povidone-iodine, physical conditions and chemical reagents. Dermatology 212:119–123. 16490989 10.1159/000089211 PMC7179540

[11] Blatchley ER, Petri B, Sun W (2021) SARS-CoV-2 ultraviolet radiation dose-response behavior. Journal of Research of the National Institute of Standards and Technology 126:126018. 38469447 10.6028/jres.126.018 PMC10857211

[12] Batoni P, Pagan JG, Harris TR, Lawal O, Linden KG, Bartow C, Beck S (2012) Early adoption of UV-C light emitting diode technology for water disinfection. *IUVA News* 14(3):18–22. Available at https://uvsolutionsmag.com/stories/pdf/archives/140301BatoniEtAl.pdf

[13] Harris TR, Pagan JG, Batoni P, and Krause JR (2014) Apparatus for irradiation. U.S. Patent Application 14/102,969, filed June 12.

[14] Sun P, Qie S, Liu Z, Ren J, Li K, Xi J (2020) Clinical characteristics of hospitalized patients with SARS-CoV-2 infection: A single arm meta-analysis. Journal of Medical Virology 92(6):612–617. 32108351 10.1002/jmv.25735 PMC7228255

[15] Duan, SM, Zhao XS, Wen RF, Huang JJ, Pi GH, Zhang SX, Han J, Bi SL, Ruan L, Dong XP, SARS Research Team (2003) Stability of SARS coronavirus in human specimens and environment and its sensitivity to heating and UV irradiation. Biomedical and Environmental Sciences 16(3):246–255. 14631830

[16] Tsunetsugu-Yokota Y (2008) Large-scale preparation of UV-inactivated SARS coronavirus virions for vaccine antigen. *SARS- and Other Coronaviruses*, ed Cavanagh D (Humana Press, Totowa, NJ) Methods in Molecular Biology (Methods and Protocols), Vol. 454. 10.1007/978-1-59745-181-9_11 PMC7122600 19057880

[17] Gallais F, Velay A, Nazon C, Wendling M, Partisani M, Sibilia J, Candon S, Fafi-Kremer S (2021) Intrafamilial exposure to SARS-CoV-2 associated with cellular immune response without seroconversion, France. Emerging Infectious Diseases 27(1):113–121. 33261718 10.3201/eid2701.203611 PMC7774579

[18] Lindsley WG, Martin SB, Thewlis RE, Sarkisian K, Nwoko JO, Mead KR, Noti JD (2015) Effects of ultraviolet germicidal irradiation (UVGI) on N95 respirator filtration performance and structural integrity. Journal of Occupational and Environmental Hygiene 12(8):509–517. 25806411 10.1080/15459624.2015.1018518 PMC4699414

[19] Mills D, Harnish DA, Lawrence C, Sandoval-Powers M, Heimbuch BK (2018) Ultraviolet germicidal irradiation of influenza-contaminated N95 filtering facepiece respirators. American Journal of Infection Control 46(7):e49–e55. 29678452 10.1016/j.ajic.2018.02.018 PMC7115285

[20] Lopez GU, Gerba CP, Tamimi AH, Kitajima M, Maxwell SL, Rose JB (2013) Transfer efficiency of bacteria and viruses from porous and nonporous fomites to fingers under different relative humidity conditions. Applied and Environmental Microbiology 79(18):5728–5734. 23851098 10.1128/AEM.01030-13 PMC3754157

[21] LightTech LightSources (2015) Germicidal Lamp Basics. Available at https://www.light-sources.com/

[22] Inagaki H, Saito S, Sugiyama H, Okabayashi T, Fujimoto S (2020) Rapid inactivation of SARS-CoV-2 with deep-UV LED irradiation. Emerging Microbes & Infections 9(1):1744-1747. 32673522 10.1080/22221751.2020.1796529 PMC7473214

[23] Pendyala B, Patras A, Pokharel B, D’Souza D (2020) Genomic modeling as an approach to identify surrogates for use in experimental validation of SARS-CoV-2 and HuNoV inactivation by UV-C treatment. Frontiers in Microbiology 11:572331. 33133042 10.3389/fmicb.2020.572331 PMC7550400

[24] Patterson EI, Prince T, Anderson ER, Casas-Sanchez A, Smith SL, Cansado-Utrilla C, Solomon T, Griffiths MJ, Acosta-Serrano A, Turtle L, Hughes GL (2020) Methods of inactivation of SARS-CoV-2 for downstream biological assays, The Journal of Infectious Diseases 222(9):1462–1467. 32798217 10.1093/infdis/jiaa507 PMC7529010

[25] Biasin M, Bianco A, Pareschi G, Cavalleri A, Cavatorta C, Fenizia C, Galli P, Lessio L, Lualdi, M, Tombetti E, Ambrosi A, Radelli EMA, Saulle I, Trabattoni D, Zanutta A, Clerici M (2021) UV-C irradiation is highly effective in inactivating SARS-CoV-2 replication. Scientific Reports 11:6260. 33737536 10.1038/s41598-021-85425-w PMC7973506

[26] Simmons S, Carrion R Jr, Alfson K, Staples H, Jinadatha C, Jarvis W, Sampathkumar P, Chemaly RF, Khawaja F, Povroznik M, Jackson S, Kaye KS, Rodriguez RM, Stibich M (2020) Disinfection effect of pulsed xenon ultraviolet irradiation on SARS-CoV-2 and implications for environmental risk of COVID-19 transmission. medRxiv*.* 10.1017/ice.2020.399 PMC7443558 32741425

